# Cyclooxygenase-2 overexpression is common in serrated and non-serrated colorectal adenoma, but uncommon in hyperplastic polyp and sessile serrated polyp/adenoma

**DOI:** 10.1186/1471-2407-8-33

**Published:** 2008-01-29

**Authors:** Takako Kawasaki, Katsuhiko Nosho, Mutsuko Ohnishi, Yuko Suemoto, Jonathan N Glickman, Andrew T Chan, Gregory J Kirkner, Mari Mino-Kenudson, Charles S Fuchs, Shuji Ogino

**Affiliations:** 1 Department of Medical Oncology, Dana-Farber Cancer Institute, Boston, MA, USA; 2 Department of Pathology, Brigham and Women's Hospital, and Harvard Medical School, Boston, MA, USA; 3 Gastrointestinal Unit, Massachusetts General Hospital, Boston, MA, USA; 4 Channing Laboratory, Department of Medicine, Brigham and Women's Hospital, and Harvard Medical School, Boston, MA, USA; 5 Department of Pathology, Massachusetts General Hospital, and Harvard Medical School, Boston, MA, USA; 6 Department of Epidemiology, Harvard School of Public Health, Boston, MA, USA

## Abstract

**Background:**

Cyclooxygenase-2 (COX-2, *PTGS2*) plays an important role in colorectal carcinogenesis. COX-2 overexpression in colorectal cancer is inversely associated with microsatellite instability (MSI) and the CpG island methylator phenotype (CIMP). Evidence suggests that MSI/CIMP+ colorectal cancer may arise through the serrated tumorigenic pathway through various forms of serrated neoplasias. Therefore, we hypothesized that COX-2 may play a less important role in the serrated pathway.

**Methods:**

By immunohistochemistry, we assessed COX-2 expression in 24 hyperplastic polyps, 7 sessile serrated polyp/adenomas (SSA), 5 mixed polyps with SSA and adenoma, 27 traditional serrated adenomas, 515 non-serrated adenomas (tubular adenoma, tubulovillous adenoma and villous adenoma), 33 adenomas with intramucosal carcinomas, 96 adenocarcinomas with serration (corkscrew gland) and 111 adenocarcinomas without serration.

**Results:**

Strong (2+) COX-2 overexpression was more common in non-serrated adenomas (28% = 143/515) than in hyperplastic polyps (4.2% = 1/24, p = 0.008) and serrated polyps (7 SSAs and 5 mixed polyps) (0% = 0/12, p = 0.04). Furthermore, any (1+/2+) COX-2 overexpression was more frequent in non-serrated adenomas (60% = 307/515) than in hyperplastic polyps (13% = 3/24, p < 0.0001) and serrated polyps (SSAs and mixed polyps) (25% = 3/12, p = 0.03). Traditional serrated adenomas and non-serrated adenomas showed similar frequencies of COX-2 overexpression. Regardless of serration, COX-2 overexpression was frequent (~85%) in colorectal adenocarcinomas. Tumor location was not significantly correlated with COX-2 overexpression, although there was a trend towards higher frequencies of COX-2 overexpression in distal tumors (than proximal tumors) among hyperplastic polyps, SSAs, mixed polyps, traditional serrated adenomas and adenocarcinomas.

**Conclusion:**

COX-2 overexpression is infrequent in hyperplastic polyp, SSA and mixed polyp with SSA and adenoma, compared to non-serrated and serrated adenoma. COX-2 overexpression becomes more frequent as tumors progress to higher grade neoplasias. Our observations suggest that COX-2 may play a less significant role in the serrated pathway of tumorigenesis; however, COX-2 may still play a role in later stage of the serrated pathway.

## Background

Cyclooxygenase-2 (COX-2 or *PTGS2*, the HUGO-approved official gene symbol) has been considered to have an important role in the development of various cancers, including colorectal cancer [1-4]. COX-2 overexpression is observed in approximately 70–80% of colorectal cancer [5-7], and has been associated with poor prognosis in some but not all studies [5,6]. Regular use of COX inhibitor aspirin has been shown to decrease risks of colorectal cancer [8] and adenoma [9], and epidemiologic evidence supports that aspirin prevents colorectal cancer by inhibiting COX-2 [10]. In addition, COX-2 selective inhibitor celecoxib inhibits the growth of colorectal cancer cells in vitro [11,12]. Randomized trials have demonstrated that celecoxib decrease a risk of recurrent adenomas in high-risk individuals [13-15]. Thus, COX-2 is a promising chemopreventive target against colorectal neoplasia [1,16,17]. In light of these observations, it may be important to examine COX-2 expression levels in precursor lesions to predict effectiveness of chemoprevention by COX-2 inhibition [10,18].

Serrated colorectal neoplasias comprise a family of lesions bearing some histological similarities, including an overall serrated configuration of neoplastic epithelial cells [19,20]. Serrated colorectal neoplasias include hyperplastic polyps, sessile serrated polyps/adenomas (SSAs), polyps with mixed features of SSA and adenoma, and traditional serrated adenomas. Accumulating evidence suggest that SSAs and polyps with mixed features of SSA and adenoma be precursor lesions for colorectal cancers, in particular, with *BRAF *mutation, high degree of MSI and widespread promoter methylation referred to as the CpG island methylator phenotype (CIMP) [19-23]. Thus, the term ''the serrated pathway'' has been used for multistep colorectal carcinogenesis through serrated precursor lesions [22-24].

COX-2 overexpression in colorectal cancer is inversely associated with MSI [25-27] and CIMP [27,28]. COX-2 has been shown to be frequently overexpressed in serrated adenomas [29,30], but infrequently in hyperplastic polyps [2,29-31]. COX-2 overexpression has been demonstrated in polyps in hereditary mixed polyposis syndrome [32] and familial juvenile polyposis [33], but not in fibroblastic polyps [34]. However, to our knowledge, no study has comprehensively examined COX-2 expression levels in various serrated and non-serrated colorectal neoplasias. Therefore, in this study, we have evaluated COX-2 expressions in various serrated and non-serrated colorectal neoplasias, including hyperplastic polyps, SSAs, traditional serrated adenomas, and adenocarcinomas with or without serration.

## Methods

### Study group

In order to recruit patients into this study, we utilized the databases of two large prospective cohort studies: the Nurses' Health Study (N = 121,700 women) [35], and the Health Professional Follow-up Study (N = 51,500 men) [36]. Informed consent was obtained from all participants prior to inclusion in the cohorts. A subset of the cohort participants developed colorectal polyps or colorectal cancers during prospective follow-up. Previous studies confirmed that our colorectal polyps/adenomas and colorectal cancers were representative as population-based samples [8,10,35-37]. We requested paraffin embedded tissue samples of colorectal polyps that were endoscopically 1 cm or greater. Polyps endoscopically measuring less than 1 cm did not give reliable results for COX-2 expression because those small polyps became even much smaller after formalin fixation and careful histopathologic examination for diagnosis at multiple levels of sections (i.e., typically only approximately 1 mm of tissue was left). Tumors were selected based on availability of tumor tissue samples and assay results at the time of this study. As a result, a total of 611 colorectal polyp cases (290 from the men's cohort and 321 from the women's cohort) were included in this study (mean age 60.8 years old, median 62, standard deviation 8.2). For the purpose of comparison, 207 colorectal adenocarcinomas from the two cohorts were also evaluated for glandular serration and COX-2 expression. Tissue collection and analyses were approved by the Dana-Farber/Harvard Cancer Center and Brigham and Women's Hospital Institutional Review Boards.

### Histopathologic evaluations

**Figure 1:**
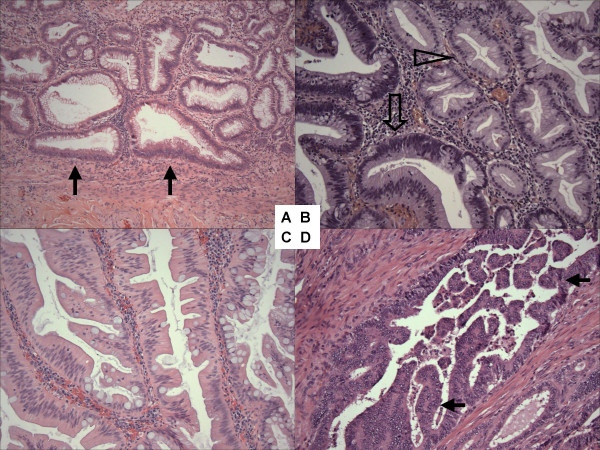
Histopathology of serrated colorectal neoplasias. A. Sessile serrated polyp/adenoma (SSA) with abnormal crypt architecture (flat-based crypt) (arrow). B. Mixed polyp with SSA (empty arrowhead) and non-serrated adenoma (empty arrow). C. traditional serrated adenoma with abundant eosinophilic cytoplasm of epithelial cells. D. Colorectal adenocarcinoma with serration (arrows).

Hematoxylin and eosin (HE) stained slides of the tumors were reviewed by a pathologist (S.O.), and colorectal neoplasias were classified according to the previously described criteria [19,38]: hyperplastic polyp, sessile serrated polyp/adenoma (SSA), polyp with mixed features of SSA and non-serrated adenoma (herein referred to as "mixed polyp"), traditional serrated adenoma (Figure 1), non-serrated adenomas (tubular adenoma, tubulovillous adenoma, and villous adenoma), and adenoma with focal intramucosal adenocarcinoma. All serrated polyps and adenomas were reviewed by a second pathologist (M.M.) and discrepant diagnoses were resolved by discussion. By definition, tubular adenoma contains villous architecture in <25% of adenomatous epithelium. Tubulovillous adenoma and villous adenoma have between 25% and 75%, and greater than 75% of villous epithelium, respectively. Serration in colorectal adenocarcinoma was evaluated according to the previously published criteria [39,40]. Similar to hyperplastic polyps, serrated colorectal polyps and traditional serrated adenomas, serrated adenocarcinomas were characterized by the presence of serrated epithelial cells projecting into the lumen of neoplastic glands (Figure 1). Glandular serration in colorectal cancer was evaluated by a pathologist (J.N.G.).

### Immunohistochemistry for COX-2

**Figure 2:**
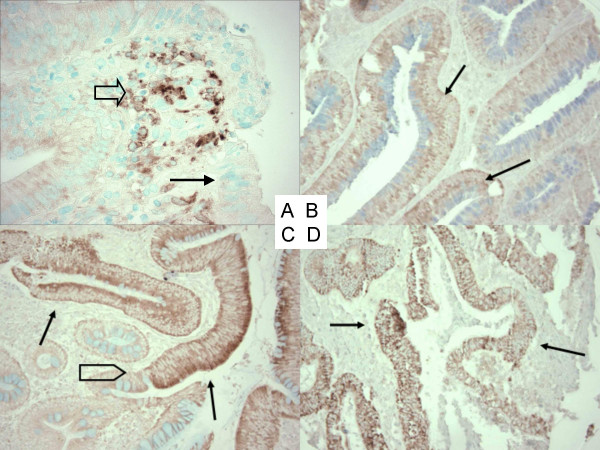
Immunohistochemistry for COX-2 in colorectal neoplasias. A. No overexpression in colorectal adenoma (arrow). Inflammatory cells serve as an internal positive control (empty arrow). B. Weak (1+) overexpression in colorectal adenoma (arrows). C. Strong (2+) overexpression in colorectal adenoma (arrows). Note a junction between adenoma and normal appearing colon (empty block arrow). D. Strong (2+) overexpression in colorectal adenocarcinoma (arrows).

Methods for COX-2 immunohistochemistry were previously described [7,10]. Briefly, antigen retrieval was performed by incubating deparaffinized tissue sections in citrate buffer (BioGenex, San Ramon, CA, USA) by a microwave for 15 min. Tissue sections were incubated with 3% H_2_O_2 _(20 min), then with Avidin Block (Vector Laboratories, Burlingame, CA, USA) (15 min), and then with Biotin Block (Vector Laboratories) (15 min). Primary anti-COX-2 antibody (Cayman Chemical, Ann Arbor, MI, USA) (dilution 1:300) was applied overnight at 4°C. Then, tissue sections were incubated with secondary anti-mouse antibody (Vector Laboratories) (20 min), and then with avidin-biotin complex conjugate (Vector Laboratories). Tissue sections were visualized by diamino benzidine (5 min) and methyl-green counterstain. Tissue microarrays (TMAs) were constructed as previously described [27], and used for immunohistochemistry in only colorectal cancer cases. For cases in which results from TMAs were equivocal, we stained whole tissue sections to obtain more definitive results. COX-2 expression in adenoma/polyp cells was interpreted as negative (no overexpression), weak overexpression (1+), or strong overexpression (2+) (Figure 2). COX-2 expression in adenomas and polyps was often heterogeneous and we interpreted as overexpression even if staining of tumor cells was focal. Inflammatory cells served as an internal positive control. Appropriate positive control (colorectal cancer with known COX-2 overexpression) and negative control (colorectal cancer with known COX-2 overexpression treated by water instead of anti-COX-2 antibody) were included in each run of immunohistochemistry. COX-2 expression in colorectal cancer was interpreted as previously described [10]. All immunohistochemically stained slides were reviewed by a pathologist (S.O.) blinded from clinical or other laboratory data. A subset of cancer cases (N = 108) was reviewed for COX-2 expression independently by two pathologists and the concordance rate was 92% (kappa coefficient = 0.62, p < 0.001) [10].

### Statistical analysis

Chi-square test (or Fisher's exact test when the number of cases in any category was less than 10) was performed for categorical data, and kappa coefficient to evaluate concordance of interpretation of COX-2 by the two observers was computed, using the SAS program (version 9.1, SAS Institute, Cary, NC). All p values were two-sided, and statistical significance was set as p ≤ 0.05.

## Results

### COX-2 overexpression is infrequent in hyperplastic polyp, sessile serrated polyp/adenoma (SSA) and mixed polyp with SSA and non-serrated adenoma

There were totals of 605 colorectal polyps and adenomas, including 24 hyperplastic polyps (HPs), 7 sessile serrated polyp/adenomas (SSAs), 5 mixed polyps (MPs) with SSA and non-serrated adenoma, 27 traditional serrated adenomas (SAs), 393 tubular adenomas, 109 tubulovillous adenomas, 13 villous adenomas, and 33 (non-serrated) adenomas with intramucosal adenocarcinoma. For the purpose of comparison, we also evaluated 207 colorectal adenocarcinomas; 96 with at least focal serration and 111 without serration. Among all 605 colorectal polyps and adenomas, weak (1+) COX-2 overexpression was observed in 191 cases (32%), and strong (2+) COX-2 overexpression was observed in 160 cases (26%). There was no significant difference in the frequencies of COX-2 overexpression between tubular adenomas, tubulovillous adenomas and villous adenomas (Table 1). Therefore, we combined these adenomas into "non-serrated adenomas".

**Table 1:** Frequency of COX-2 overexpression in colorectal tumors

Tumor type	Total	COX-2 negative	COX-2 weak (1+) overexpression	COX-2 strong (2+) overexpression	COX-2 any (1+, 2+) overexpression
Hyperplastic polyp (HP)	24	21 (88%)	2 (8.3%)	1 (4.2%)	3 (13%)
Sessile serrated polyp/adenoma (SSA)	7	5 (71%)	2 (29%)	0	2 (29%)
Mixed polyp with SSA and adenoma (MP)	5	4 (80%)	1 (20%)	0	1 (20%)
Total HP, SSA and MP	36	30 (83%)	5 (14%)	1 (2.8%)	6 (17%)
Traditional serrated adenoma	27	8 (30%)	14 (52%)	5 (19%)	19 (70%)
Tubular adenoma (TA)	393	154 (39%)	132 (34%)	107 (27%)	239 (61%)
Tubulovillous adenoma (TVA)	109	49 (45%)	27 (25%)	33 (30%)	60 (55%)
Villous adenoma (VA)	13	5 (38%)	5 (38%)	3 (23%)	8 (62%)
Total non-serrated adenomas (TA, TVA, and VA)	515	208 (40%)	164 (32%)	143 (28%)	307 (60%)
Adenoma with intramucosal carcinoma	33	8 (24%)	11 (33%)	14 (42%)	25 (76%)
Adenocarcinoma with serration	96	13 (14%)	18 (19%)	65 (68%)	83 (86%)
Adenocarcinoma without serration	111	18 (16%)	23 (21%)	70 (63%)	93 (84%)
Total adenocarcinomas	207	31 (15%)	41 (20%)	135 (65%)	176 (85%)

**Figure 3:**
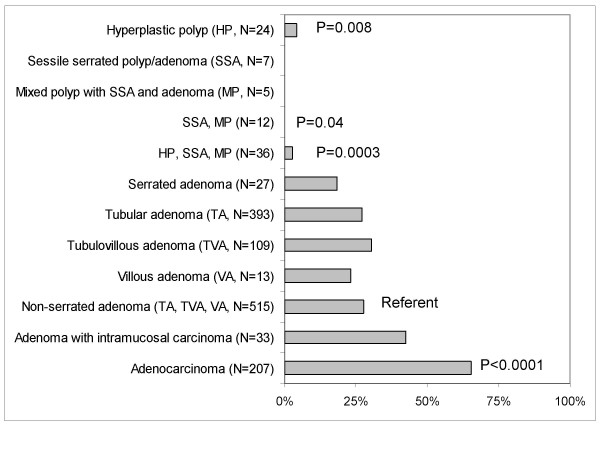
Frequency of strong (2+) COX-2 overexpression in various colorectal neoplasias.

**Figure 4:**
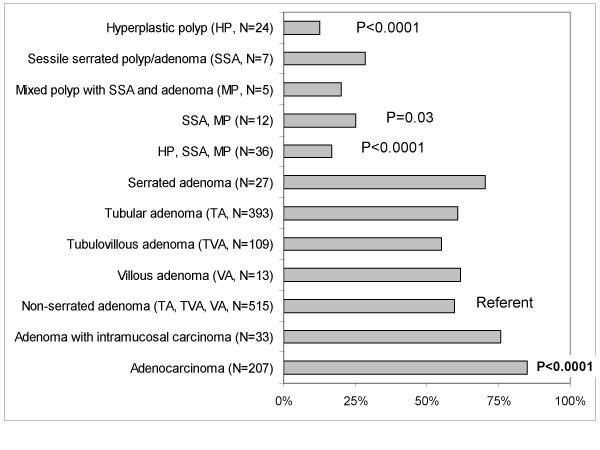
Frequency of any (1+ or 2+) COX-2 overexpression in various colorectal neoplasias.

Compared to non-serrated adenomas, COX-2 expression levels were generally lower in hyperplastic polyps, sessile serrated adenomas (SSAs), and mixed polyps with SSA and adenoma. In contrast to non-serrated adenomas [showing 2+ COX-2 in 28% (143/515) of cases], strong (2+) COX-2 expression was observed in none (0%) of 12 serrated lesions including 7 SSAs and 5 mixed polyps (p = 0.04) and in only 4.2% (1/24) of hyperplastic polyps (p = 0.008) (Table 1, Figure 3). Any (1+, 2+) COX-2 expression was more common in non-serrated adenomas (60% = 307/515) than in hyperplastic polyps (13% = 3/24, p < 0.0001), and serrated lesions including SSAs and mixed polyps (25% = 3/12, p = 0.03) (Table 1, Figure 4). Despite the presence of serrated appearance, traditional serrated adenomas showed the frequency of COX-2 overexpression similar to non-serrated adenomas. We noted that most hyperplastic polyps were small (typically <3 mm on glass slides) although these were supposed to be endoscopically ≥ 1 cm. Since we interpreted focal staining as positivity, the proportion of negative hyperplastic polyps might be merely function of size. Nonetheless, focal COX-2 positivity was considered to be important, because focal positive cells might progress to COX-2 positive advanced lesions.

### COX-2 overexpression was not correlated with serration in colorectal adenocarcinoma

In adenocarcinomas, regardless of the presence or absence of serration, COX-2 overexpression was frequent (Table 1). Strong (2+) COX-2 overexpression was more common in adenocarcinomas (65% = 135/207) than in non-serrated adenomas (28% = 143/515, p < 0.0001) (Figure 3). Any (1+ or 2+) COX-2 overexpression was also more common in adenocarcinomas (85% = 176/207) than in non-serrated adenomas (60% = 307/515, p < 0.0001) (Figure 4). There was a trend that COX-2 overexpression was progressively more common from non-serrated adenomas, to adenomas with intramucosal carcinomas, and to adenocarcinomas.

### Tumor location, gender, age at diagnosis, and COX-2 overexpression in colorectal neoplasias

We also examined the relationship between tumor location, histopathologic diagnosis and the frequency of COX-2 overexpression (Table 2). There were no significant differences in the frequencies of COX-2 overexpression between tumors in the proximal colon and those in the distal colorectum in any histopathologic category. Nonetheless, there was a trend towards more frequent COX-2 overexpression in tumors in the distal colorectum than those in the proximal colon (among hyperplastic polyps, SSAs, mixed polyps, traditional serrated adenomas and adenocarcinomas).

COX-2 overexpression was not correlated with gender or age at diagnosis in any of the histopathologic categories examined (data not shown).

**Table 2:** Frequency of COX-2 overexpression in colorectal tumors according to tumor location

Tumor type	Total	COX-2 negative	COX-2 weak (1+) overexpression	COX-2 strong (2+) overexpression	COX-2 any (1+, 2+) overexpression
Proximal*					
Hyperplastic polyp (HP)	5	5 (100%)	0	0	0
Sessile serrated polyp/adenoma (SSA)	2	2 (100%)	0	0	0
Mixed polyp with SSA and adenoma (MP)	1	1 (100%)	0	0	0
Traditional serrated adenoma	8	4 (50%)	3 (38%)	1 (13%)	4 (50%)
Tubular adenoma (TA)	120	53 (44%)	47 (39%)	20 (17%)	67 (56%)
Tubulovillous adenoma (TVA)	32	11 (34%)	9 (28%)	12 (38%)	21 (66%)
Villous adenoma (VA)	3	1 (33%)	1 (33%)	1 (33%)	2 (67%)
Adenoma with intramucosal carcinoma	7	3 (43%)	1 (14%)	3 (43%)	4 (57%)
Adenocarcinoma with serration	34	6 (18%)	7 (21%)	21 (62%)	28 (82%)
Adenocarcinoma without serration	31	8 (26%)	7 (23%)	16 (52%)	23 (74%)
Distal*					
Hyperplastic polyp (HP)	17	14 (82%)	2 (12%)	1 (5.9%)	3 (28%)
Sessile serrated polyp/adenoma (SSA)	4	2 (50%)	2 (50%)	0	2 (50%)
Mixed polyp with SSA and adenoma (MP)	3	2 (67%)	1 (33%)	0	1 (33%)
Traditional serrated adenoma	19	4 (21%)	11 (58%)	4 (21%)	15 (79%)
Tubular adenoma (TA)	192	76 (40%)	57 (30%)	59 (31%)	116 (60%)
Tubulovillous adenoma (TVA)	65	35 (54%)	16 (25%)	14 (22%)	30 (46%)
Villous adenoma (VA)	9	4 (44%)	3 (33%)	2 (22%)	5 (56%)
Adenoma with intramucosal carcinoma	12	2 (17%)	5 (42%)	5 (42%)	10 (83%)
Adenocarcinoma with serration	34	3 (8.8%)	4 (12%)	27 (79%)	31 (91%)
Adenocarcinoma without serration	42	4 (9.5%)	9 (21%)	29 (69%)	38 (90%)

* Proximal tumors were located from cecum to transverse colon, and distal tumors were located from splenic flexure to rectum.

## Discussion

We conducted this study to examine COX-2 expression in various serrated and non-serrated colorectal neoplasias. We have demonstrated that, compared to non-serrated adenomas, COX-2 overexpression is infrequent in hyperplastic polyps, sessile serrated polyps/adenomas (SSAs) and mixed polyps with SSA and adenoma. Traditional serrated adenoma and non-serrated adenoma show similar frequencies of COX-2 overexpression. In addition, COX-2 overexpression appears to become frequent as tumors progress to higher grade neoplasias, which is in agreement with a previous study showing a positive correlation between COX-2 expression and tumor grade in colorectal adenomas [41].

SSAs have been named in a variety of ways in the literature [19]. Torlakovic and Snover [42] found that the histopathologic features of polyps of "hyperplastic polyposis" were different from those of small sporadic hyperplastic polyps, and the term "sessile serrated adenoma" was coined to distinguish these distinct lesions from the more pedunculated lesions of traditional serrated adenomas [43]. Similar lesions have been reported under different names, including "serrated adenoma, superficial types" [44] and "serrated adenoma types 1 and 2" [45]. A recent review by Jass [23] used the term "sessile serrated polyp" because "this lesion lacks the traditional cytology of colorectal adenoma and in order to avoid confusion with serrated adenoma, it is referred to in this review as sessile serrated polyp". To reconcile this terminology issue, we used the term "sessile serrated polyp/adenoma (SSA)".

A few previous studies have examined COX-2 expressions in sporadic serrated adenomas as well as hyperplastic polyps. Arao et al. [30] examined COX-2 expression in serrated adenomas and hyperplastic polyps, and found that 71% of serrated adenomas showed moderate to intense positivity, in contrast to 32% of hyperplastic polyps showing weak to moderate positivity. Takeuchi et al. [29] examined COX-2 expression in serrated adenomas, tubular adenomas and hyperplastic polyps, but not in SSAs. They found that serrated adenomas of the cerebriform pattern showed a similar COX-2 expression score as tubular adenomas, and concluded that serrated adenoma of the cerebriform pattern should be treated similarly as tubular adenoma. There are other studies that have shown infrequent COX-2 overexpression in hyperplastic polyps [2,31]. However, to our knowledge, no study to date has compared COX-2 expression levels between SSAs, traditional serrated adenomas, non-serrated adenomas and adenocarcinomas.

The serrated pathway of colorectal carcinogenesis has been linked to widespread promoter methylation referred to as the CpG island methylator phenotype (CIMP) [23,46]. CIMP-high colorectal cancers are inversely correlated with COX-2 overexpression independent of MSI status [27]. Together with our current data on the inverse association between COX-2 and the serrated polyps, these observations support the proposed link between the serrated pathway and CIMP-high in colorectal cancer development.

Our data may have significant clinical implications because of emerging importance of COX-2 as a promising chemopreventive target [10,18]. Regular aspirin use has been shown to decrease risks of colorectal cancer as well as adenoma [8,9]. We have recently shown that regular aspirin use decreases a risk of COX-2-overexpressing colorectal cancer, but not a risk of COX-2-negative colorectal cancer [10]. In light of our findings, aspirin, celecoxib, and other non-steroidal anti-inflammatory drugs (NSAIDs) may be less frequently effective against serrated polyps than non-serrated adenomas. However, since COX-2 overexpression also appears to be dependent on tumor progression, it is possible that COX-2 inhibition may still be effective for the prevention of tumor progression in a subset of any types of precursor lesions.

## Conclusion

In conclusion, hyperplastic polyp, sessile serrated polyp/adenoma (SSA) and mixed polyp with SSA and adenoma infrequently overexpress COX-2, when compared to non-serrated adenomas and serrated adenomas. COX-2 overexpression appears to be more frequent as tumors progress. Our observations suggest that COX-2 may play a less significant role in the serrated pathway of tumorigenesis, especially in early stage of polyp formation; however, COX-2 may still play a role in later stage of the serrated pathway.

## List of abbreviations

COX-2: Cyclooxygenase-2 (PTGS2, the HUGO Gene Nomenclature Committee-approved official gene symbol); HP: Hyperplastic polyp; MP: Mixed polyp; SSA: Sessile serrated polyp/adenoma; TA: Tubular adenoma; TVA: Tubulovillous adenoma; VA: Villous adenoma.

## Competing interests

The author(s) declare that they have no competing interests.

## Authors' contributions

TK, KN and MO performed the assays, analyzed the data and drafted the manuscript. YS performed and interpreted the assays. JNG interpreted morphology of carcinomas and interpreted the data. ATC interpreted the data. GJK coordinated the study, and analyzed and interpreted the data. MM interpreted morphology of polyps/adenomas as a second pathologist, interpreted the data. CSF coordinated the study, discussed the study design and data. SO conceived the study, designed assays, analyzed and interpreted the data, and drafted the manuscript. All authors read and approved the final manuscript.

## Pre-publication history

The pre-publication history for this paper can be accessed here:



## References

[B1] Brown JR, DuBois RN (2005). COX-2: a molecular target for colorectal cancer prevention. J Clin Oncol.

[B2] Sheehan KM, O'Connell F, O'Grady A, Conroy RM, Leader MB, Byrne MF, Murray FE, Kay EW (2004). The relationship between cyclooxygenase-2 expression and characteristics of malignant transformation in human colorectal adenomas. Eur J Gastroenterol Hepatol.

[B3] Larkins TL, Nowell M, Singh S, Sanford GL (2006). Inhibition of cyclooxygenase-2 decreases breast cancer cell motility, invasion and matrix metalloproteinase expression. BMC Cancer.

[B4] Klenke FM, Gebhard MM, Ewerbeck V, Abdollahi A, Huber PE, Sckell A (2006). The selective Cox-2 inhibitor Celecoxib suppresses angiogenesis and growth of secondary bone tumors: an intravital microscopy study in mice. BMC Cancer.

[B5] Fux R, Schwab M, Thon KP, Gleiter CH, Fritz P (2005). Cyclooxygenase-2 expression in human colorectal cancer is unrelated to overall patient survival. Clin Cancer Res.

[B6] Soumaoro LT, Uetake H, Higuchi T, Takagi Y, Enomoto M, Sugihara K (2004). Cyclooxygenase-2 expression: a significant prognostic indicator for patients with colorectal cancer. Clin Cancer Res.

[B7] Ogino S, Brahmandam M, Cantor M, Namgyal C, Kawasaki T, Kirkner G, Meyerhardt JA, Loda M, Fuchs CS (2006). Distinct molecular features of colorectal carcinoma with signet ring cell component and colorectal carcinoma with mucinous component. Mod Pathol.

[B8] Giovannucci E, Egan KM, Hunter DJ, Stampfer MJ, Colditz GA, Willett WC, Speizer FE (1995). Aspirin and the risk of colorectal cancer in women [see comments]. N Engl J Med.

[B9] Giovannucci E, Rimm EB, Stampfer MJ, Colditz GA, Ascherio A, Willett WC (1994). Aspirin use and the risk for colorectal cancer and adenoma in male health professionals [see comments]. Ann Intern Med.

[B10] Chan AT, Ogino S, Fuchs CS (2007). Aspirin and the Risk of Colorectal Cancer in Relation to the Expression of COX-2. New Engl J Med.

[B11] Kazanov D, Dvory-Sobol H, Pick M, Liberman E, Strier L, Choen-Noyman E, Deutsch V, Kunik T, Arber N (2004). Celecoxib but not rofecoxib inhibits the growth of transformed cells in vitro. Clin Cancer Res.

[B12] Lev-Ari S, Strier L, Kazanov D, Madar-Shapiro L, Dvory-Sobol H, Pinchuk I, Marian B, Lichtenberg D, Arber N (2005). Celecoxib and curcumin synergistically inhibit the growth of colorectal cancer cells. Clin Cancer Res.

[B13] Steinbach G, Lynch PM, Phillips RK, Wallace MH, Hawk E, Gordon GB, Wakabayashi N, Saunders B, Shen Y, Fujimura T, Su LK, Levin B (2000). The effect of celecoxib, a cyclooxygenase-2 inhibitor, in familial adenomatous polyposis. N Engl J Med.

[B14] Arber N, Eagle CJ, Spicak J, Racz I, Dite P, Hajer J, Zavoral M, Lechuga MJ, Gerletti P, Tang J, Rosenstein RB, Macdonald K, Bhadra P, Fowler R, Wittes J, Zauber AG, Solomon SD, Levin B (2006). Celecoxib for the prevention of colorectal adenomatous polyps. N Engl J Med.

[B15] Bertagnolli MM, Eagle CJ, Zauber AG, Redston M, Solomon SD, Kim K, Tang J, Rosenstein RB, Wittes J, Corle D, Hess TM, Woloj GM, Boisserie F, Anderson WF, Viner JL, Bagheri D, Burn J, Chung DC, Dewar T, Foley TR, Hoffman N, Macrae F, Pruitt RE, Saltzman JR, Salzberg B, Sylwestrowicz T, Gordon GB, Hawk ET (2006). Celecoxib for the prevention of sporadic colorectal adenomas. N Engl J Med.

[B16] Dannenberg AJ, Lippman SM, Mann JR, Subbaramaiah K, DuBois RN (2005). Cyclooxygenase-2 and epidermal growth factor receptor: pharmacologic targets for chemoprevention. J Clin Oncol.

[B17] Samoha S, Arber N (2005). Cyclooxygenase-2 inhibition prevents colorectal cancer: from the bench to the bed side. Oncology.

[B18] Markowitz SD (2007). Aspirin and colon cancer--targeting prevention?. N Engl J Med.

[B19] Snover DC, Jass JR, Fenoglio-Preiser C, Batts KP (2005). Serrated polyps of the large intestine: a morphologic and molecular review of an evolving concept. Am J Clin Pathol.

[B20] Torlakovic E, Snover DC (2006). Sessile serrated adenoma: a brief history and current status. Crit Rev Oncog.

[B21] Kambara T, Simms LA, Whitehall VLJ, Spring KJ, Wynter CVA, Walsh MD, Barker MA, Arnold S, McGivern A, Matsubara N, Tanaka N, Higuchi T, Young J, Jass JR, Leggett BA (2004). BRAF mutation is associated with DNA methylation in serrated polyps and cancers of the colorectum. Gut.

[B22] Jass JR (2003). Serrated adenoma of the colorectum: a lesion with teeth. Am J Pathol.

[B23] Jass JR (2005). Serrated adenoma of the colorectum and the DNA-methylator phenotype. Nat Clin Pract Oncol.

[B24] Jass JR (2001). Serrated route to colorectal cancer: back street or super highway?. J Pathol.

[B25] Sinicrope FA, Lemoine M, Xi L, Lynch PM, Cleary KR, Shen Y, Frazier ML (1999). Reduced expression of cyclooxygenase 2 proteins in hereditary nonpolyposis colorectal cancers relative to sporadic cancers. Gastroenterology.

[B26] Karnes WE, Shattuck-Brandt R, Burgart LJ, DuBois RN, Tester DJ, Cunningham JM, Kim CY, McDonnell SK, Schaid DJ, Thibodeau SN (1998). Reduced COX-2 protein in colorectal cancer with defective mismatch repair. Cancer Res.

[B27] Ogino S, Brahmandam M, kawasaki T, Kirkner GJ, Loda M, Fuchs CS (2006). Combined analysis of COX-2 and p53 expressions reveals synergistic inverse correlations with microsatellite instability and CpG island methylator phenotype in colorectal cancer. Neoplasia.

[B28] Toyota M, Shen L, Ohe-Toyota M, Hamilton SR, Sinicrope FA, Issa JP (2000). Aberrant methylation of the Cyclooxygenase 2 CpG island in colorectal tumors. Cancer Res.

[B29] Takeuchi M, Kobayashi M, Ajioka Y, Honma T, Suzuki Y, Azumaya M, Narisawa R, Hayashi S, Asakura H (2002). Comparison of cyclo-oxygenase 2 expression in colorectal serrated adenomas to expression in tubular adenomas and hyperplastic polyps. Int J Colorectal Dis.

[B30] Arao J, Sano Y, Fujii T, Kato S, Fu KI, Yoshino T, Ochiai A, Fujimori T, Yoshida S (2001). Cyclooxygenase-2 is overexpressed in serrated adenoma of the colorectum. Dis Colon Rectum.

[B31] Maekawa M, Sugano K, Sano H, Miyazaki S, Ushiama M, Fujita S, Gotoda T, Yokota T, Ohkura H, Kakizoe T, Sekiya T (1998). Increased expression of cyclooxygenase-2 to -1 in human colorectal cancers and adenomas, but not in hyperplastic polyps. Jpn J Clin Oncol.

[B32] Brazowski E, Misonzhnick-Bedny F, Rozen P (2004). Cyclooxygenase-2 expression in the hereditary mixed polyposis syndrome. Dig Dis Sci.

[B33] Brazowski E, Rozen P, Misonzhnick-Bedny F, Gitstein G (2005). Characteristics of familial juvenile polyps expressing cyclooxygenase-2. Am J Gastroenterol.

[B34] Groisman GM, Polak-Charcon S, Appelman HD (2006). Fibroblastic polyp of the colon: clinicopathological analysis of 10 cases with emphasis on its common association with serrated crypts. Histopathology.

[B35] Colditz GA, Hankinson SE (2005). The Nurses' Health Study: lifestyle and health among women. Nat Rev Cancer.

[B36] Wei EK, Giovannucci E, Fuchs CS, Willett WC, Mantzoros CS (2005). Low Plasma Adiponectin Levels and Risk of Colorectal Cancer in Men: A Prospective Study. J Natl Cancer Inst.

[B37] Giovannucci E, Colditz GA, Stampfer MJ, Willett WC (1996). Physical activity, obesity, and risk of colorectal adenoma in women (United States). Cancer Causes Control.

[B38] Hamilton SR, Vogelstein B, Kudo S, Aaltonen LA, Hamilton SR (2000). Carcinoma of the colon and rectum. Pathology and genetics of tumours of the digestive system World Health Organization classification of tumours.

[B39] Makinen MJ (2007). Colorectal serrated adenocarcinoma. Histopathology.

[B40] Chirieac LR, Shen L, Catalano PJ, Issa JP, Hamilton SR (2005). Phenotype of microsatellite-stable colorectal carcinomas with CpG island methylation. Am J Surg Pathol.

[B41] Sato T, Yoshinaga K, Okabe S, Okawa T, Enomoto M, Takizawa T, Sugihara K (2003). Cyclooxygenase-2 expression in colorectal adenomas. Dis Colon Rectum.

[B42] Torlakovic E, Snover DC (1996). Serrated adenomatous polyposis in humans. Gastroenterology.

[B43] Torlakovic E, Skovlund E, Snover DC, Torlakovic G, Nesland JM (2003). Morphologic reappraisal of serrated colorectal polyps. Am J Surg Pathol.

[B44] Oka S, Tanaka S, Hiyama T, Ito M, Kitadai Y, Yoshihara M, Haruma K, Chayama K (2004). Clinicopathologic and endoscopic features of colorectal serrated adenoma: differences between polypoid and superficial types. Gastrointest Endosc.

[B45] Mitomi H, Sada M, Kobayashi K, Igarashi M, Mori A, Kanazawa H, Nishiyama Y, Ihara A, Otani Y (2003). Different apoptotic activity and p21(WAF1/CIP1), but not p27(Kip1), expression in serrated adenomas as compared with traditional adenomas and hyperplastic polyps of the colorectum. J Cancer Res Clin Oncol.

[B46] Minoo P, Jass J (2006). Senescence and serration: a new twist to an old tale. J Pathol.

